# Role and Mechanism of Short-Chain Fatty Acids in Skeletal Muscle Homeostasis and Exercise Performance

**DOI:** 10.3390/nu17091463

**Published:** 2025-04-26

**Authors:** Xiaoguang Liu, Miaomiao Xu, Huiguo Wang, Lin Zhu

**Affiliations:** 1 School of Sport and Health, Guangzhou Sport University, Guangzhou 510500, China; liuxg@gzsport.edu.cn (X.L.); 11450@gzsport.edu.cn (H.W.); 2 School of Physical Education and Health, Guangzhou University of Chinese Medicine, Guangzhou 510006, China; miaomiaoxu@gzucm.edu.cn

**Keywords:** short-chain fatty acids, exercise performance, skeletal muscle, metabolism, inflammation, oxidative stress

## Abstract

Acetate, propionate, and butyrate are major short-chain fatty acids (SCFAs) produced by the gut microbiota as key metabolic byproducts. These SCFAs influence skeletal muscle homeostasis and exercise performance through various mechanisms. This review explores the current knowledge on the mechanisms by which SCFAs influence muscle mass, strength, metabolism, and inflammation. Acetate enhances mitochondrial function and glucose metabolism, while butyrate supports muscle mass preservation by suppressing inflammation and autophagy. Propionate plays complex roles, aiding metabolic regulation, but potentially impairing myogenic differentiation at high concentrations. These SCFAs modulate insulin sensitivity and oxidative stress; their interactions with host energy systems and immunity significantly influence muscle health. Although their therapeutic potential is evident, further studies, especially human clinical trials, are necessary to validate their effective applications. This review synthesizes emerging evidence and outlines specific future research priorities.

## 1. Introduction

Skeletal muscle homeostasis is a dynamic process that maintains the balance between muscle protein synthesis and degradation, which is crucial for muscle mass and function [1,2]. This balance is critical for the proper functioning of skeletal muscles, the largest organ in the human body, which plays a pivotal role in whole-body energy metabolism [3,4]. Skeletal muscle homeostasis relies on the dynamic interplay between protein synthesis and degradation. An increase in synthesis promotes hypertrophy, while decreased synthesis and heightened degradation result in muscle atrophy and, under certain pathological conditions, wasting [5].

Exercise and nutrition are key modulators of skeletal muscle homeostasis [6]. External stimuli—including exercise, hormonal fluctuations, changes in oxygen and nutrient availability, and motor neuron activity—regulate the structural and functional characteristics of skeletal muscle. Exercise, particularly resistance and endurance training, enhances muscle protein synthesis, muscle function, and performance. However, these effects depend significantly on exercise type, intensity, volume, nutritional intake, and recovery strategies [7,8].

As we understand more about the regulation of skeletal muscle function and mass, it is clear that factors beyond traditional muscle synthesis and degradation play significant roles. One of the most intriguing aspects of muscle health involves short-chain fatty acids (SCFAs), metabolites produced by gut microbiota that have a profound impact on muscle homeostasis.

SCFAs, generated through gut microbial fermentation of non-digestible carbohydrates, are absorbed from the intestinal lumen and influence metabolic processes across multiple organs, including skeletal muscle [9,10]. Notably, SCFAs like acetate, propionate, and butyrate impact skeletal muscle metabolism—affecting lipids, carbohydrates, and proteins—and contribute significantly to muscle function and exercise performance [11,12,13]. Recent studies have highlighted the pivotal role of gut microbiota-derived SCFAs, particularly acetate, propionate, and butyrate, in modulating skeletal muscle function. For example, Yang et al. [14] demonstrated that acetate supplementation alleviated muscle growth retardation in germ-free mice, underlining the critical role of SCFAs in maintaining muscle mass through microbiota-derived metabolites. Additionally, recent reviews such as Frampton et al. [12] have explored how SCFAs improve mitochondrial function, enhance oxidative metabolism, and modulate muscle hypertrophy, making them potential therapeutic agents for muscle-related disorders.

Unlike previous reviews, this manuscript provides an integrated analysis of SCFAs, specifically focusing on their differential and concentration-dependent effects on muscle mass, function, metabolism, autophagy, oxidative stress, and inflammation. Additionally, we discuss practical implications for therapeutic and athletic applications, emphasizing areas that require further investigation through clinical studies.

Beyond their established metabolic roles, SCFAs have been shown to directly influence muscle mass and function in diverse populations. This review explores their contributions to skeletal muscle homeostasis and exercise performance, a topic of growing scientific and practical relevance. Understanding these mechanisms holds promise for advancing health, athletic performance, and public health strategies. Continued research is vital to unlock the full therapeutic potential of SCFAs in enhancing muscle function.

## 2. Materials and Methods

### 2.1. Literature Search Strategy

A systematic search was conducted using PubMed and Web of Science databases between 1 January 2005 and 15 April 2025. Keywords included “short-chain fatty acids”, “skeletal muscle”, “exercise performance”, “metabolism”, “inflammation”, and “oxidative stress”.

Additionally, reference lists of identified articles were manually checked to uncover any potentially overlooked studies.

### 2.2. Inclusion and Exclusion Criteria

Studies were selected based on the following inclusion criteria: (1) publication in peer-reviewed journals, (2) English language, (3) direct relevance to short-chain fatty acids in skeletal muscle homeostasis and exercise performance, and (4) presentation of original data or comprehensive reviews. Excluded were non-English publications, conference abstracts, and studies unrelated to the core topic. The initial search yielded 2482 articles, of which 2432 were excluded based on the exclusion criteria, leaving 50 articles for full-text review.

## 3. Results

### 3.1. Short-Chain Fatty Acids: Types and Sources

Within the complex human gut ecosystem, SCFAs—including acetate, propionate, and butyrate—play crucial roles in regulating metabolic balance and diverse physiological functions. Besides these, valerate and caproate are less-abundant SCFAs found at lower concentrations in the gut. Their specific biological roles are less well-characterized. Dietary fibers, which are abundant in fruits, vegetables, legumes, and whole grains, are primary sources for SCFA production (Table 1). However, modern diets, often low in dietary fiber and high in processed foods, can significantly diminish SCFA production, potentially contributing to issues in muscle and metabolic health.

Acetate is the most abundant SCFA in the human gut, comprising around 60% of total SCFA production [21]. It is primarily generated through the fermentation of dietary fibers by gut microbes such as Bacteroides and Faecalibacterium. Functionally, acetate contributes to lipid metabolism by acting as a substrate for de novo lipogenesis, thereby supporting energy storage and influencing body fat composition [22]. It also affects cholesterol homeostasis by modulating hepatic synthesis and the mobilization of lipid stores [23]. Beyond metabolic roles, acetate participates in appetite regulation by promoting the secretion of hormones like glucagon-like peptide-1 (GLP-1) and peptide YY (PYY), which suppress food intake and support energy balance [13].

Propionate is primarily produced through the microbial fermentation of soluble fibers by bacteria like Bacteroides and Lactobacillus [24]. Propionate is significant in gluconeogenesis, serving as a substrate for glucose synthesis in the liver, thus helping to modulate blood glucose levels and improve insulin sensitivity [25,26]. It may also inhibit hepatic cholesterol synthesis and promote cholesterol excretion, potentially reducing the risk of cardiovascular disease [27,28,29]. Additionally, propionate exhibits anti-inflammatory properties that help regulate immune activity in the gut [30].

Butyrate accounts for approximately 10–20% of total SCFA production in the colon and is chiefly derived from the fermentation of dietary fibers, especially resistant starch. Key butyrate-producing microbes include Faecalibacterium prausnitzii and Roseburia intestinalis [31]. As the primary energy source for colonocytes, butyrate supports cellular metabolism and enhances epithelial integrity, which is crucial for overall gut health. It also reinforces the intestinal barrier to prevent pathogen translocation and exerts potent anti-inflammatory effects by downregulating pro-inflammatory cytokines [32]. Additionally, butyrate modulates gene expression by inhibiting histone deacetylases (HDACs), thereby influencing pathways linked to inflammation and metabolism [33].

### 3.2. Role and Mechanisms of SCFAs in Skeletal Muscle Homeostasis and Exercise Performance

These mechanistic insights into SCFAs suggest their significant influence on skeletal muscle health, particularly regarding muscle mass and function. In this context, recent studies have shown that SCFAs can both promote muscle hypertrophy and alleviate muscle atrophy.

#### 3.2.1. SCFAs and Muscle Mass and Exercise Performance

The association between SCFAs and skeletal muscle health is evident across diverse populations, with research indicating a clear link between higher SCFA levels and improved muscle mass and function. For instance, children with elevated levels of fecal butyric acid, acetic acid, and total SCFAs showed greater total body lean soft tissue mass (TSM) and appendicular skeletal muscle mass (ASM), TSM/height^2^ (TSMI), ASM/height^2^ (ASMI), and ASMI Z-score and lower TSM/total body mass (TBF), ASM/AFM, TSM/weight (TSMR), ASM/weight (ASMR), and ASMR Z-score [34]. These findings indicate that the relationship between gut microbiota, SCFAs, and skeletal muscle quality in children may be largely influenced by total body fat content. This relationship is not limited to children; similar findings have been reported in menopausal women. For example, Lv et al. [35] dentified a positive correlation between the gut microbial synthesis of butyrate and both serum butyrate levels and the skeletal muscle index (SMI) in Chinese Han menopausal women. Notably, the correlation between butyrate and SMI was statistically significant (Spearman correlation coefficient = 0.084, *p* = 0.002), indicating that SCFAs, particularly butyrate, play a role in muscle preservation during aging [35]. These findings suggest that SCFAs derived from the gut microbiota have a widespread effect on skeletal muscle health. While these studies highlight associations between SCFA levels and skeletal muscle quality, it is crucial to consider the potential confounding factors, such as dietary patterns and lifestyle habits. Future studies that control for these variables are necessary to confirm direct causal effects. In addition, the relationship between SCFAs and muscle mass may be influenced by factors such as total body fat content, as seen in children. Future research should explore these dynamics further to clarify the specific mechanisms by which SCFAs help modulate muscle function across different populations.

Beyond promoting muscle growth, SCFAs also demonstrate potential in reversing muscle atrophy and in enhancing exercise performance. Particularly, studies on SCFA supplementation have shown promising results in preventing or alleviating muscle wasting. Specifically, SCFA-treated (sodium acetate 67.5 mM, sodium butyrate 40 mM, and sodium propionate 25.9 mM, daily treatment for 3 months) mice exhibited an increased muscle fiber cross-sectional area (CSA) and enhanced muscle strength, as evidenced by improved grip strength and ex vivo muscle function tests in pre-sarcopenic senescent accelerated mouse prone 8 (SAMP8) mice. Additionally, SCFAs enhanced muscle endurance, as demonstrated by improved performance in treadmill tests, indicating better anti-fatigue capacity in SAMP8 mice [36]. Consistent with these results, butyrate-treated db/db mice significantly blocked the decrease in gastrocnemius muscle mass and CSA compared with control mice [37]. In a murine model of cachexia (C26 tumor-bearing male BALB/c mice), butyrate supplementation (once every 24 h at a dose of 200 mg/kg) significantly attenuated muscle atrophy. Mice treated with butyrate showed reduced weight loss, improved grip strength, and increased muscle mass (tibialis anterior (TA), gastrocnemius (GA), and extensor digitorum brevis (EDL)) compared to untreated cachectic mice [38]. Furthermore, oral acetate supplementation in antibiotic-treated mice prevents the reduction in grip strength and muscle fiber CSA, which is associated with decreased intestinal SCFA concentrations, including acetate [38]. Mice treated with antibiotics for two weeks exhibited significantly reduced grip strength and smaller cross-sectional area (CSA) of muscle fibers, indicating impaired muscle performance. This effect was associated with a decrease in intestinal SCFA concentrations, including acetate. Oral acetate supplementation (150 mM sodium acetate solution) in antibiotic-treated mice prevented the reduction in grip strength and muscle fiber CSA. Moreover, Acetyl-CoA synthase 2 (AceCS2) knockout mice, which are unable to utilize acetate efficiently, exhibited reduced skeletal muscle mass, smaller muscle fiber CSA, and shorter lifespan compared to wild-type mice [39]. In conclusion, acetate derived from the gut microbiota is essential for maintaining skeletal muscle mass and strength in mice. It acts as an important energy substrate, and may influence muscle performance through its metabolic actions. Corroborating these observations, in a HG/LPS- induced C2C12 myotube atrophy model, the butyrate treatment significantly prevented myotube atrophy [37].

In summary, SCFAs, particularly acetate, propionate, and butyrate, significantly enhance exercise performance and muscle health. SCFA supplementation has been shown to prevent muscle wasting and improve muscle mass, strength, and endurance in animal models. Specifically, supplementation increased muscle fiber cross-sectional area, improved grip strength, and enhanced treadmill endurance performance, indicating reduced fatigue. Additionally, SCFAs, notably butyrate and acetate, have demonstrated protective effects against muscle atrophy and functional decline in conditions like cachexia and antibiotic-induced gut microbiota disruption. These benefits underscore the essential role of gut microbiota-derived SCFAs in maintaining muscle mass and optimizing exercise performance.

SCFA is not only involved in skeletal muscle atrophy and development, but also in muscle hypertrophy. Acetate (1.97 mg/mL) was administered to mice undergoing mechanical overload-induced muscle hypertrophy [40]. In the early stages of mechanical overload-induced hypertrophy, acetate did not significantly enhance muscle fiber cross-sectional area (CSA). However, after 5 days, acetate treatment was shown to significantly increase mitochondrial respiration, suggesting a role in supporting muscle energetics during hypertrophy. Specifically, acetate elevated complex I and complex II respiration rates, indicating enhanced oxidative metabolism [40]. This suggests that acetate may support muscle energetics during hypertrophy by improving mitochondrial function. However, the evidence is mixed, and further research is needed to validate these findings in human populations. Future studies should focus on randomized controlled trials to elucidate the long-term effects of SCFA supplementation and optimize dosing strategies for clinical applications.

Skeletal muscle development is also influenced by SCFAs. Germ-free (GF) mice supplemented with sodium acetate (150 mmol/L) exhibit increased serum acetate levels, improved body weight gain, and enhanced SDH activity compared to vehicle (water) GF mice. The expression of myogenic regulatory factors (MRFs), particularly Mef2a, is upregulated in the GS group, suggesting that acetate can mitigate the negative effects of gut microbiota depletion on skeletal muscle development [14]. Supporting these results, acetate treatment enhances the expression of myogenic markers (Mef2a, Myod1, Myog) and promotes myotube fusion in C2C12 skeletal muscle cells [14]. However, not all SCFAs contribute positively to skeletal muscle development. Propionate may impair skeletal muscle development by inhibiting myogenic differentiation. Extracellular propionate, but not acetate, significantly inhibits the differentiation of C2C12 myoblasts into myotubes and similarly disrupts this process in primary human muscle cells [41]. This is evidenced by the lack of myosin heavy chain (MHC) expression, a marker of differentiated myotubes, in cells exposed to propionate. The mechanism underlying propionate-impaired myogenic differentiation involves increasing histone propionylation and acetylation, which may dysregulate the normal transcriptional control of myogenic genes [41]. These results suggest that not all SCFAs contributed skeletal muscle development, and that propionate may impair skeletal muscle development in C2C12 myoblasts. However, further studies are still needed to explore other SCFAs in skeletal muscle development. In addition, the larger animals model or randomized controlled trial still need in further to test the effects of SCFAs for skeletal muscle development.

In summary, SCFAs significantly impact skeletal muscle mass and exercise performance. Research across diverse populations—including children and menopausal women—has revealed positive associations between SCFA levels and improved muscle mass and function, suggesting that gut microbiota-derived SCFAs broadly support skeletal muscle health. Animal studies further confirm these benefits, demonstrating that SCFA supplementation effectively prevents muscle wasting, enhances muscle fiber size, improves strength, and increases endurance capacity. Mechanistically, SCFAs contribute to muscle health by promoting mitochondrial energy metabolism, reducing fatigue, and protecting against muscle atrophy caused by cachexia or disruptions in gut microbiota. However, SCFA effects can vary; for instance, while acetate supports muscle energetics and hypertrophy, propionate at higher concentrations can negatively affect muscle differentiation. Future studies, particularly randomized controlled human trials, are needed to further clarify these relationships and optimize the clinical use of SCFAs for enhancing muscle health and exercise performance.

#### 3.2.2. SCFAs and Skeletal Muscle Homeostasis

##### SCFAs and Protein Synthesis and Degradation

SCFAs, particularly butyrate, have been shown to activate the mammalian target of the rapamycin (mTOR) signaling pathway, a key regulator of protein synthesis. This mechanism enhances muscle protein synthesis, promoting hypertrophy in skeletal muscle, as demonstrated in senescent accelerated mouse models [36]. Acetate supplementation was reported to upregulate myogenic regulatory factors (e.g., Myod1, Myog), enhancing myoblast differentiation and protein synthesis in germ-free mice models [14]. Besides autophagy inhibition, butyrate modulates muscle degradation by reducing inflammation-driven proteolysis. It decreases inflammatory cytokines, such as TNF-α and NF-κB, which are involved in promoting muscle proteolysis [42]. Additionally, SCFAs have been associated with the downregulation of the ubiquitin–proteasome pathway, another crucial system responsible for muscle protein degradation, further protecting against muscle wasting observed in cachexia and metabolic disease models [38]. In summary, SCFAs, notably butyrate and acetate, enhance skeletal muscle protein synthesis via mTOR pathway activation and the upregulation of myogenic regulatory factors (Myod1, Myog). They also suppress muscle degradation by reducing inflammation-driven proteolysis and downregulating the ubiquitin–proteasome pathway, thereby protecting against muscle-wasting conditions.

##### SCFAs and Skeletal Muscle Metabolism

In addition to modulating muscle mass, SCFAs also play a significant role in skeletal muscle metabolism. Skeletal muscle metabolism and skeletal muscle homeostasis are intricately linked, with metabolism providing the energy and substrates necessary for maintaining muscle function and structure, while homeostasis ensures the balance and stability of muscle tissue in response to various physiological and pathological conditions.

SCFAs improve intramuscular lipid metabolism. There was lower serum butyric acid in SAMP8 sarcopenic mice as compared with control mice (senescence-accelerated mouse resistant 1). SCFAs daily treatment (sodium acetate 67.5 mM, sodium butyrate 40 mM, and sodium propionate 25.9 mM in water) decreased fat infiltration in skeletal muscle in SAMP8 sarcopenic mice models as compared with the vehicle group (sodium water) [36]. This effect was associated with an increased expression of genes related to lipid oxidation (e.g., PPARδ) in skeletal muscle as compared with sodium water treatment, indicating that SCFAs enhance fatty acid oxidation and reduce lipid accumulation in muscle tissue [36]. In addition to the combined effects of SCFAs, individual SCFAs also exhibit significant benefits. For instance, in a PCOS rat model, acetate (oral gavage, 200 mg/kg in water, for 21 days) treatment significantly decreases the levels of triglycerides (TG), total cholesterol (TC), and free fatty acids (FFA) in skeletal muscle as compared with vehicle group (water), suggesting that it can mitigate lipid accumulation and lipotoxicity in insulin-resistant skeletal muscle [42]. Acetic acid treatment also increases the phosphorylation of AMPK and the expression of GLUT4, myoglobin, and myocyte enhancer factor 2A (MEF2A) in the L6 rat skeletal myoblast cell line. Moreover, compared with the vehicle group, acetic acid treatment increased glucose and fatty acid uptake and decreased triglyceride accumulation in L6 myotube cells [43]. In summary, SCFAs—such as acetate—promote intramuscular lipid metabolism by boosting fatty acid oxidation and limiting lipid buildup in skeletal muscle. This is evidenced by the decreased fat infiltration and increased expression of lipid oxidation-related genes (e.g., PPARδ) in sarcopenic mice, as well as reduced levels of triglycerides, total cholesterol, and free fatty acids in PCOS rat models. Additionally, acetic acid promotes glucose and fatty acid uptake and activates AMPK and GLUT4 in skeletal muscle cells, further supporting its role in mitigating lipotoxicity and improving muscle metabolic health. Although acetic acid and acetate salts (such as sodium acetate) share similar metabolic pathways, differences in absorption rates, local gastrointestinal effects, and systemic availability might occur. Further comparative studies are needed to clearly define their bioequivalence.

SCFAs improve skeletal muscle insulin resistance. In addition to improving intramuscular lipid metabolism, SCFAs are also involved in regulating skeletal muscle insulin resistance. Skeletal muscle insulin resistance is often associated with severe obesity and type 2 diabetes. Mice supplemented with butyrate (sodium butyrate incorporated with high-fat diet (58% calories from fat) at 5% wt/wt) showed prevention of diet-induced obesity and insulin resistance as compared with high-fat diet mice. In addition, it enhanced mitochondrial function and biogenesis by increasing the expression of PGC-1α in brown adipose tissue (BAT) and skeletal muscle [44]. Moreover, butyrate enhances insulin sensitivity and stimulates glycolysis in C2C12 cells without necessitating increased long-chain fatty acid oxidation. Its catabolism acts as a regulatory mechanism that mitigates HDAC inhibition. Consequently, blocking butyrate oxidation indirectly prevents insulin resistance and promotes glycolytic activity in myotubes, likely through a HDAC-dependent pathway [45]. These data indicate that butyrate improves skeletal muscle insulin resistance through enhancing mitochondrial function and biogenesis. The findings are supported by high-quality in vivo and in vitro studies. However, the long-term effects of butyrate supplementation in humans remain unclear, and further research is needed to elucidate the precise molecular pathways involved.

Propionate, which has primarily been studied as inulin–propionate ester or propionic acid, has been associated with improved insulin sensitivity in various models. Dietary supplementation with inulin–propionate ester (IPE) and inulin significantly enhanced insulin sensitivity compared to cellulose, a low-fermentable fiber control, as assessed by the homeostatic model assessment 2 (HOMA2-IR) and the Matsuda insulin sensitivity index. The improvements were driven by a significant reduction in fasting insulin levels [26]. In an in vitro experiment, propionic acid (300 µM) increased both basal and insulin-stimulated glucose uptake in C2C12 myotubes, with a 12.4% increase in basal uptake and a 26.4% increase in insulin-stimulated uptake [46]. A 5 mM mixture of acetate, propionate, and butyrate in a 60:20:20 ratio significantly enhanced glucose uptake in C2C12 myotubes [47]. In contrast, elevated levels of propionate and butyrate (20 mM) impaired insulin-stimulated glucose uptake [47]. This may be due to the cytotoxicity associated with high concentrations of propionate in skeletal muscle cells. While the results are promising, high concentrations of propionate (20 mM) decreased insulin-dependent glucose uptake, potentially due to cytotoxicity [47]. This suggests a concentration-dependent effect, and further studies are needed to determine the optimal therapeutic concentration. Furthermore, more direct studies using pure propionate salts are necessary for definitive conclusions.

Acetate also contributes to improved glucose homeostasis and insulin sensitivity in skeletal muscle. In a PCOS rat model, acetate treatment elevated both glycogen content and glycogen synthase activity compared to the vehicle group [42]. This is crucial for maintaining glucose homeostasis and reducing the risk of insulin resistance. Consistent with these findings, long-term acetate administration (5.2 mg/kg in water, 5 days per week for 6 months) upregulated the expression of myoglobin and GLUT4 genes in the abdominal muscle of Otsuka Long-Evans Tokushima Fatty (OLETF) rats compared to vehicle-treated controls [48]. The OLETF rat is a spontaneous type 2 diabetes mellitus (T2DM) model rat with characteristics of obesity, hyperphagia, diabetes, and metabolic disorders. The findings suggest a beneficial role for acetate in improving glucose homeostasis. However, the precise mechanisms driving these effects remain unclear, and their relevance to human physiology warrants further exploration.

In summary, SCFAs, including butyrate, propionate, and acetate, collectively improve skeletal muscle insulin resistance through multiple mechanisms. Butyrate enhances insulin sensitivity and mitochondrial function, while propionate significantly boosts glucose uptake and reduces fasting insulin levels. Acetate also increases glycogen content and GLUT4 expression, contributing to better glucose homeostasis. However, high concentrations of propionate may exhibit cytotoxicity and impair insulin-dependent glucose uptake. These findings highlight the potential of SCFAs as therapeutic agents for mitigating insulin resistance and associated metabolic disorders in conditions such as obesity and type 2 diabetes.

##### SCFAs Inhibited Skeletal Muscle Autophagy and Oxidative Stress

Autophagy is a key factor in skeletal muscle homeostasis. SCFAs regulate the number of autophagosomes to maintain skeletal muscle homeostasis. In a randomized controlled trial involving a mouse model of diabetic nephropathy-induced skeletal muscle atrophy, butyrate (1 g/kg/day in diet) treatment significantly decreased the number of autophagosomes and autolysosomes in skeletal muscle as compared with standard diet mice [37]. Moreover, butyrate treatment significantly inhibits the upregulation of LC3II and the downregulation of p62 in skeletal muscle and HG/LPS-induced cell models [37]. In addition, targeted metabolomics analyses found that compared with healthy subjects, there were 146 metabolites in serum found as compared with diabetic nephropathy patients. The butyrate metabolism pathway was significantly enriched [37]. The findings are supported by both in vivo and in vitro studies, indicating that butyrate can modulate autophagy-related pathways. However, the study lacks a detailed mechanistic explanation of how butyrate specifically targets autophagy components. Additional studies are required to clarify the signaling pathways involved.

Oxidative stress is a key factor in skeletal muscle homeostasis. SCFAs regulate the redox balance to maintain skeletal muscle homeostasis. Butyrate (1 g/kg/day) addition in the diet significantly inhibited the decrease in catalase (CAT), glutathione peroxidase (GSH-Px), and superoxide dismutase (SOD) in a randomized controlled trial involving a mouse model of diabetic nephropathy-induced skeletal muscle atrophy [37]. The findings are supported by robust in vivo evidence, indicating that butyrate has a protective effect against oxidative stress in skeletal muscle. However, the study lacks detailed mechanistic insights into how butyrate specifically enhances antioxidant enzyme activity. Further research is needed to explore the signaling pathways involved in this process. Consistent with these findings, acetate mitigates oxidative stress and enhances antioxidant defenses in skeletal muscle. In the letrozole-induced PCOS rat model, acetate (200 mg/kg, oral gavage, 21 days) addition reduced skeletal muscle malondialdehyde (MDA) levels, a marker of lipid peroxidation, while increasing glutathione (GSH) and nuclear factor erythroid 2-related factor 2 (Nrf2) levels as compared with the vehicle (water) group [42]. Similar results were observed in ex vivo experiments. Compared with the vehicle group, acetate, propionate, or butyrate (1, 5, and 20 mM) treatment significantly increased the GSH levels in C2C12 myotubes [47]. The findings are supported by both in vivo and in vitro studies, suggesting that acetate can effectively enhance antioxidant defenses in skeletal muscle. However, the study does not provide a detailed comparison of the effects of different SCFAs (acetate, propionate, butyrate) on oxidative stress markers. Additionally, the concentration-dependent effects of these SCFAs are not fully explored, and further studies are needed to determine their optimal therapeutic concentrations.

In summary, SCFAs, including butyrate, acetate, and propionate, exhibit significant potential in modulating autophagy and oxidative stress in skeletal muscle, thereby contributing to the maintenance of skeletal muscle homeostasis under various pathological conditions. Furthermore, the consistent results across different animal models and cell lines reinforce the potential therapeutic applications of SCFAs in skeletal muscle disorders. Future studies should aim to uncover the detailed molecular mechanisms of SCFA activity and pinpoint specific targets for therapeutic intervention.

##### SCFAs Inhibited Skeletal Muscle Inflammation

Inflammation significantly influences skeletal muscle homeostasis. Beyond metabolism and muscle mass, SCFAs also address another critical factor in muscle health: inflammation. Chronic low-grade inflammation is often a contributing factor to both muscle atrophy and insulin resistance. Interestingly, SCFAs have shown the ability to reduce inflammatory markers, further supporting their role in muscle health.

SCFAs can regulate skeletal muscle homeostasis by modulating inflammation. Sarcopenic mice exhibited higher levels of lipopolysaccharide (LPS) in their bloodstream as compared with young mice, indicative of increased gut permeability. SCFA cocktail (sodium acetate (67.5 mM), sodium butyrate (40 mM), and sodium propionate (25.9 mM)) daily treatment for 3 months lowers LPS levels and enhances the expression of intestinal barrier proteins (e.g., Muc2 and Claudin1) as compared with the vehicle (water) group, thereby reducing inflammation. This systemic anti-inflammatory effect likely contributed to the overall improvement in muscle health, as chronic inflammation is a key driver of sarcopenia [36]. Consistent with the reduction in systemic inflammation by SCFAs, a randomized crossover trial found that inulin–propionate ester (IPE) supplementation (20 g/day, for 42 day) significantly decreases levels of the pro-inflammatory cytokine IL-8 as compared to cellulose supplementation in obesity and overweight adults [26]. This effect was supported by in vitro experiments showing that peripheral blood mononuclear cells (PBMCs) cultured with sodium propionate secreted less IL-8 compared to sodium acetate or sodium chloride [26]. Additionally, daily oral administration of propionate (150 mM for 4 weeks) significantly increased regulatory T cell (Treg) counts and interleukin-10 (IL-10) levels in the intestinal microenvironment of high-fat diet-fed ApoE^-^/^-^ mice, compared to vehicle treatment (0.9% sodium chloride) [30]. In summary, SCFAs play a crucial role in regulating skeletal muscle homeostasis by modulating systemic inflammation and enhancing gut barrier integrity, thereby mitigating sarcopenia and improving muscle health. The findings are supported by both in vivo and in vitro studies, indicating that SCFAs can modulate systemic inflammation through gut barrier enhancement. However, the specific mechanisms by which SCFAs regulate gut barrier integrity and inflammation remain unclear. Further research is needed to elucidate the underlying pathways and determine the long-term effects of SCFA supplementation in humans.

Moreover, SCFAs reduce local inflammation in skeletal muscle across multiple disease models. Butyrate (200 mg/kg, gavage, daily for 21 days) treatment significantly decreased the infiltration of F4/80^+^ macrophages in skeletal muscle compared with C26 tumor cells-induced cachexia BALB/c mice. Moreover, butyrate treatment significantly decreased the number of M1 macrophages and increased M2 macrophages in skeletal muscle compared with the vehicle group [38]. These findings were supported by in vitro studies showing that butyrate supplementation (1 mM for 24 h) in the culture medium suppresses LPS-induced M1 macrophage polarization while synergistically enhancing IL-4-induced M2 polarization [38]. PCOS is associated with reduced informal abnormal skeletal muscle homeostasis, including inflammation. Acetate treatment (oral gavage, 200 mg/kg in water, for 21 days) significantly decreased the levels of inflammatory biomarkers, including nuclear factor-kB (NF-kB) and tumor necrosis factor-alpha (TNF-α), and attenuated the expression of the NLRP3 inflammasome in skeletal muscle as compared with vehicle group (water) in a PCOS rat model [42]. In addition, in an obese skeletal muscle cell model (10 ng/mL lipopolysaccharide (LPS) and 500 μM palmitic acid (PA) for 24 h), a lower butyrate concentration (0.5 mM) reduced RANTES and IL-6 secretion, NFKB, and STAT3 activation. Propionate (0.5 mM) and acetate (0.5 mM) only inhibited the expression of RANTES. Higher butyrate concentration (2.5 mM) reduced IL-6, MCP-1, and RANTES secretion; higher propionate concentration (2.5 mM) reduced IL-6 and RANTES; and higher acetate concentration (2.5 mM) only reduced RANTES secretion [49]. The findings provide strong evidence that SCFAs can modulate local inflammation in skeletal muscle across multiple disease models. However, the concentration-dependent effects of SCFAs on cytokine secretion and macrophage polarization are complex and require further investigation. Additionally, the study lacks a comprehensive comparison of the efficacy of different SCFAs in modulating inflammation, and the long-term effects of SCFA treatment in human skeletal muscle remain unknown.

Given the dominant focus of the current SCFA literature on muscle hypertrophy and strength, it is important to also consider cardiorespiratory parameters. Emerging evidence, such as the NOODLE study [50], highlights the value of variables like oxygen pulse (O2Ppeak), ventilatory efficiency (VE/VCO2), oxygen uptake efficiency plateau (OUEP), and oxygen uptake efficiency slope (OUES) in assessing cardiorespiratory fitness. Future research should integrate these markers when evaluating SCFA effects on exercise performance.

**Figure 1:**
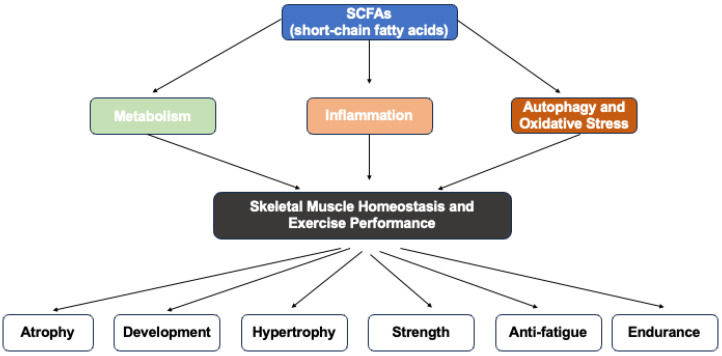
Graphical representation of this study. Key point: short-chain fatty acids (SCFAs) could modulate skeletal muscle homeostasis and exercise performance through metabolism, inflammation, autophagy and oxidative stress.

In summary, SCFAs demonstrate potent anti-inflammatory effects on skeletal muscle by modulating both systemic and local inflammation across various disease models. They reduce gut permeability, lower pro-inflammatory cytokines, and enhance regulatory immune responses. Additionally, SCFAs such as butyrate, propionate, and acetate differentially inhibit the secretion of inflammatory mediators and the activation of inflammatory pathways in skeletal muscle cells, highlighting their potential as therapeutic agents for mitigating muscle inflammation and improving muscle health in conditions like sarcopenia, cachexia, and metabolic disorders (Figure 1 and Table 2).

## 4. Conclusions

The findings from this review underscore the potential of SCFAs, particularly acetate, propionate, and butyrate, as promising therapeutic agents for treating muscle atrophy, sarcopenia, and metabolic disorders. These SCFAs improve muscle function, metabolism, and inflammation through various mechanisms, and their use in clinical settings could significantly impact muscle health and performance.

## 5. Further Studies Directions

Future work should focus on well-designed clinical trials to confirm the efficacy of SCFAs in humans. Specific attention is needed to determine optimal dosing strategies, duration of interventions, and the safety profile of SCFA supplementation across diverse populations. Moreover, integrating cardiorespiratory assessments and functional biomarkers will allow a more comprehensive understanding of SCFA benefits in exercise physiology and metabolic health.

## Figures and Tables

**Table 1:** Short-chain fatty acids: types and sources.

SCFAs	Major Fiber Sources	Key Producing Bacteria	Principal Biological Effects	References
Acetate	Resistant starch, inulin, pectin, arabinoxylan, cellulose, fructooligosaccharides (FOS)	Bifidobacterium; Bacteroides; Prevotella; Ruminococcus	Systemic energy metabolism; enhances lipid synthesis; appetite regulation via gut–brain axis; supports mucosal immunity and barrier integrity.	[15,16]
Propionate	Resistant starch, arabinoxylan, oat bran, beta-glucan, guar gum, pectin	Bacteroides; Prevotella; Veillonella; Roseburia; Dialister; Akkermansia muciniphila	Regulates hepatic gluconeogenesis; modulates lipid metabolism and cholesterol reduction; induces satiety; anti-inflammatory properties.	[16,17,18]
Butyrate	Resistant starch, inulin, pectin, psyllium husk, wheat bran, FOS	Faecalibacterium prausnitzii, Roseburia; Eubacterium rectale; Anaerostipes; Clostridium butyricum	Primary energy source for colonocytes; enhances gut barrier function and integrity; anti-inflammatory and immunomodulatory effects; inhibits colorectal cancer cell growth.	[16,19,20]

**Table 2:** SCFAs and skeletal in skeletal muscle homeostasis and exercise performance.

Objective	Methodology	Main Findings	Implications	References
Assess acetate’s effect on muscle development in germ-free mice	Acetate supplementation in germ-free mice	Improved body weight, SDH activity, and expression of myogenic factors	Acetate mitigates microbiota depletion effects on muscle development	[14]
Examine propionate’s effect on metabolic health in overweight adults	Inulin–propionate ester supplementation in a crossover trial	Improved insulin sensitivity, reduced inflammation	Propionate beneficial for metabolic improvements via SCFA pathway	[26]
Investigate SCFA levels and muscle mass in children	Fecal SCFA measurement and body composition analysis	Higher SCFA levels correlated with greater muscle mass and better muscle-to-fat ratio	SCFAs may support skeletal muscle quality in children	[34]
Examine butyrate and muscle mass in menopausal women	Cross-sectional correlation study using serum butyrate and SMI	Serum butyrate is positively associated with skeletal muscle index (SMI)	Butyrate supports muscle preservation in aging populations.	[35]
Evaluate SCFA effects in a sarcopenia mouse model	3-month treatment with SCFA cocktail in SAMP8 mice.	Increased CSA and strength, reduced fatigue.	SCFA supplementation improves muscle mass and endurance	[36]
Assess butyrate effects in diabetic nephropathy mice	Dietary butyrate intervention with muscle histology and function	Reduced autophagy markers and improved antioxidant defenses	Butyrate protects muscle via anti-autophagy and anti-oxidative mechanisms	[37]
Test butyrate in cachexia-induced muscle loss	Butyrate treatment in tumor-bearing mice	Decreased weight loss, improved strength, and anti-inflammatory response	Butyrate attenuates cachexia and improves muscle inflammation	[38]
Evaluate acetate’s role in skeletal muscle maintenance	Acetate supplementation and genetic knockout model	Improved CSA and strength; knockout mice showed reduced muscle mass	Acetate is essential for muscle mass and strength maintenance	[39]
Investigate acetate and succinate effects during muscle hypertrophy	Mechanical overload-induced hypertrophy in mice with acetate treatment	Enhanced mitochondrial respiration after 5 days	Acetate supports energetics during muscle hypertrophy	[40]
Explore propionate’s effect on myogenesis	In vitro C2C12 differentiation assay	Propionate impaired myogenic differentiation via histone modification	Not all SCFAs are beneficial; propionate may inhibit muscle development	[41]
Explore acetate’s role in a PCOS model affecting muscle	Acetate gavage in PCOS rat model	Improved glucose metabolism, decreased inflammation and oxidative stress	Acetate restores metabolic function in skeletal muscle under PCOS	[42]
Study acetic acid’s effect on AMPK activation in muscle cells	L6 myotube cells treated with acetic acid	Activated AMPK, increased glucose and FA uptake, reduced TG	Acetic acid boosts energy metabolism and reduces lipid toxicity	[43]
Assess butyrate’s impact on insulin sensitivity and energy expenditure	Butyrate incorporated into high-fat diet in mice	Prevented obesity and insulin resistance, increased PGC-1α	Butyrate supports metabolic health via enhanced mitochondrial function	[44]
Assess butyrate effects on insulin sensitivity	C2C12 myotube cultures treated with butyrate	Enhanced insulin sensitivity and glycolysis via HDAC inhibition	Butyrate improves insulin action in skeletal muscle cells	[45]
Determine SCFAs’ effect on glucose uptake in muscle cells	C2C12 myotubes treated with propionate and valerate	Increased insulin-stimulated glucose uptake via GPR41	SCFAs enhance glucose handling in skeletal muscle cells	[46]
Investigate whether SCFAs increase glucose uptake by upregulating GSH in C2C12 myotubes.	C2C12 myotubes treated with single or combined SCFAs (1, 5, 20 mM) for 24 h; glucose uptake, cytotoxicity, and GSH levels measured.	5 mM SCFA mixture increased glucose uptake; 20 mM propionate, butyrate, and mixtures reduced glucose uptake; all SCFAs increased GSH levels, but GSH increase not linked to glucose uptake; SCFAs did not prevent menadione-induced glucose uptake decrease.	Physiological levels of SCFAs can improve glucose uptake in muscle cells; however, this effect is not mediated through GSH-related antioxidant mechanisms	[47]
Explore acetate’s effect on metabolism in diabetic rats	Long-term acetate administration in OLETF rats	Increased GLUT4 and myoglobin expression, improved glucose homeostasis	Acetate improves muscle insulin sensitivity in diabetic models	[48]
Evaluate SCFAs on inflammation in muscle cells	Obese muscle cell model with SCFA treatments	SCFAs reduced pro-inflammatory cytokines in a dose-dependent manner	SCFAs modulate muscle inflammation under obese conditions	[49]

## Data Availability

Not applicable.

## Abbreviations

The following abbreviations are used in this manuscript:

SCFAs	Short-chain fatty acids
GLP-1	Glucagon-like peptide-1
PYY	Peptide YY
PPARδ	Peroxisome proliferator-activated receptor delta
AMPK	AMP-activated protein kinase
GLUT4	Glucose transporter type 4
T2DM	Type 2 diabetes mellitus
TGF-β	Transforming growth factor beta
NF-κB	Nuclear factor kappa B
MHC	Myosin heavy chain
Mef2a	Myocyte enhancer factor 2A
ACECS2	Acetyl-CoA synthase 2
HOMA2-IR	Homeostasis Model Assessment of Insulin Resistance
SAMP8	Senescence-accelerated mouse prone 8
C2C12	Mouse myoblast cell line
PCOS	Polycystic ovary syndrome
IL-8	Interleukin 8
Tregs	Regulatory T cells
RANTES	Regulated upon activation, normal T cell expressed and secreted
LPS	Lipopolysaccharide
HDAC	Histone deacetylase
NLRP3	NOD-like receptor family, pyrin domain containing 3
SCFAs	Short-chain fatty acids
